# Supplementary figures and images for: Genomic Characterization and Expression Analysis of Basic Helix-Loop-Helix (bHLH) Family Genes in Traditional Chinese Herb Dendrobium officinale
Source: Plants (Basel). 2020 Aug 17;9(8):1044. doi: 10.3390/plants9081044 (PMC7463459; doi:10.3390/plants9081044)

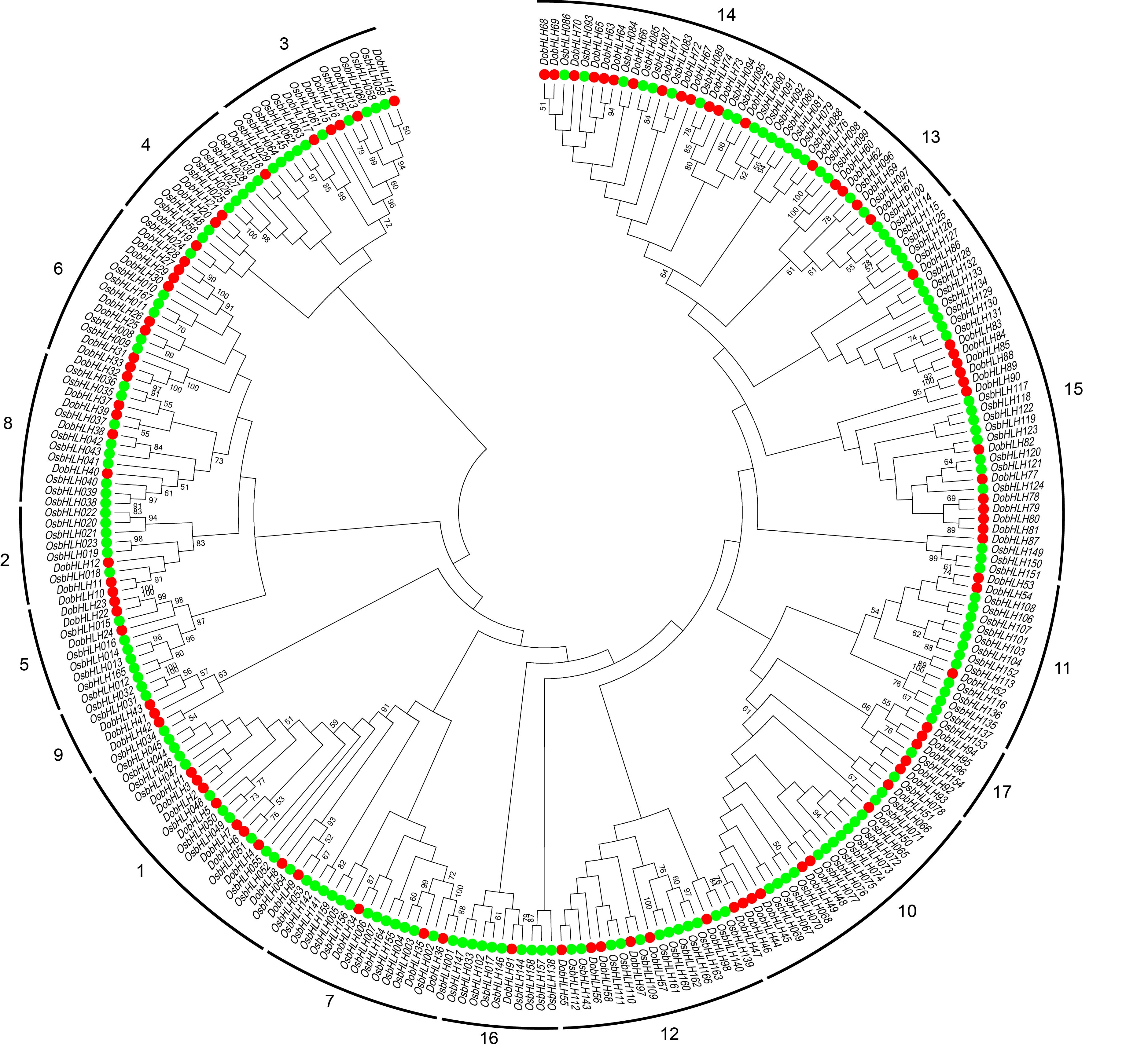

Supplement: Supplementary file 1 [file plants-09-01044-s001.zip › Supplementary files/Figure S1.jpg]

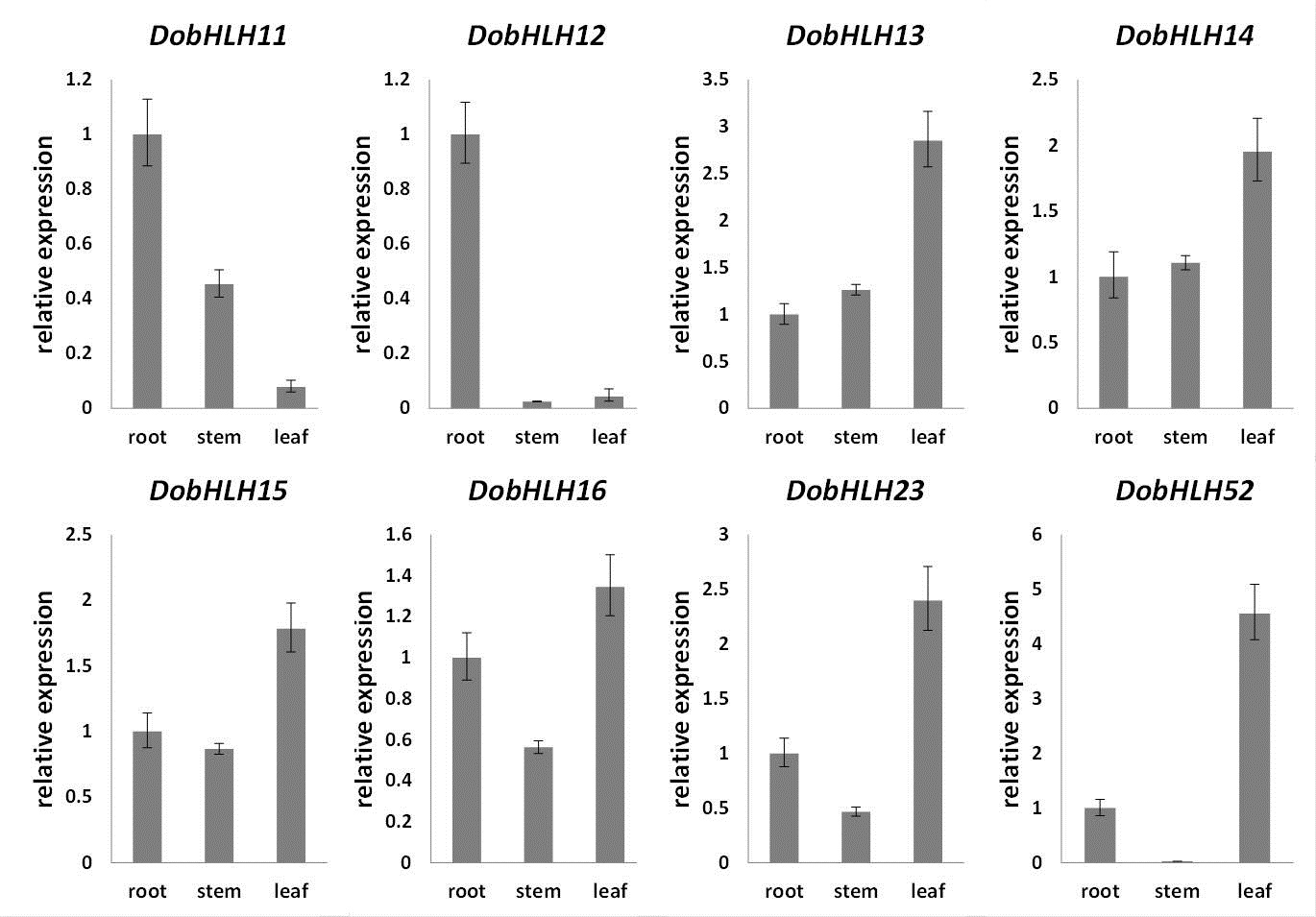

Supplement: Supplementary file 1 [file plants-09-01044-s001.zip › Supplementary files/Figure S2.jpg]
